# Parcellation of the Hippocampus Using Resting Functional Connectivity in Temporal Lobe Epilepsy

**DOI:** 10.3389/fneur.2019.00920

**Published:** 2019-08-22

**Authors:** Alexander J. Barnett, Vincent Man, Mary Pat McAndrews

**Affiliations:** ^1^Krembil Research Institute, University Health Network, Toronto, ON, Canada; ^2^Department of Psychology, University of Toronto, Toronto, ON, Canada; ^3^Division of Humanities and Social Sciences, California Institute of Technology, Pasadena, CA, United States

**Keywords:** hippocampus, epilepsy, resting state, memory, long axis, default mode network

## Abstract

We have previously shown that the connectivity of the hippocampus to other regions of the default mode network (DMN) is a strong indicator of memory ability in people with temporal lobe epilepsy (TLE). Recent work in the cognitive neuroscience literature has suggested that the anterior and posterior aspects of the hippocampus have distinct connections to the rest of the DMN and may support different memory operations. Further, structural analysis of epileptogenic hippocampi has found greater atrophy, characterized by mesial temporal sclerosis, in the anterior region of the hippocampus. Here, we used resting state FMRI data to parcellate the hippocampus according to its functional connectivity to the rest of the brain in people with left lateralized TLE (LTLE) and right lateralized TLE (RTLE), and in a group of neurologically healthy controls. We found similar anterior and posterior compartments in all groups. However, there was weaker connectivity of the epileptogenic hippocampus to multiple regions of the DMN. Both TLE groups showed reduced connectivity of the posterior hippocampus to key hubs of the DMN, the posterior cingulate cortex (PCC) and the medial pre-frontal cortex (mPFC). In the LTLE group, the anterior hippocampus also showed reduced connectivity to the DMN, and this effect was influenced by the presence of mesial temporal sclerosis. When we explored brain-behavior relationships, we found that reduced connectivity of the left anterior hippocampus to the DMN hubs related to poorer verbal memory ability in people with LTLE, and reduced connectivity of the right posterior hippocampus to the PCC related to poorer visual memory ability in those with RTLE. These findings may inform models regarding functional distinctions of the hippocampal anteroposterior axis.

## Introduction

Resting state functional connectivity has emerged as a potentially valuable tool for interrogating system integrity and predicting treatment outcome in neurological and psychiatric disease populations (1, 2). Evidence from our group and others has demonstrated that resting connectivity among default mode network (DMN) nodes is altered in temporal lobe epilepsy (TLE) (3–5), is useful for characterizing memory network integrity (4, 5), and is useful for predicting pre- to post-operative memory change (6) following surgery for TLE. TLE surgery typically involves unilateral resection of the hippocampus, amygdala, and a varying extent of anterior temporal neocortex. Specifically, the epileptogenic hippocampus, considered the site of seizure generation, is consistently reported to have reduced connectivity with major hubs of the DMN, such as the posterior cingulate cortex (PCC) and medial pre-frontal cortex (mPFC) (3, 4, 7)

Recent work in the cognitive neuroscience literature has highlighted a distinction between the anterior and posterior hippocampus in terms of their roles in cognition (8–10) and in terms of network connectivity (11–13). The anterior hippocampus has preferential connectivity to the temporal pole, perirhinal cortex, and mPFC while the posterior hippocampus has biased connections to parahippocampus, fusiform gyrus, and PCC (11–13). The mPFC and PCC are known to be critical hubs for the DMN (14) and, given the biased connectivity along the long axis of the hippocampus, critical network properties may be missed if the hippocampus is treated as a homogenous region of interest. This long-axis distinction is of further importance because structural atrophy in the hippocampus is thought to be biased in people with TLE, with greater atrophy occurring in the head of the hippocampus compared to the body and tail measured on MRI (15), measured post-mortem (16), and on resected tissues (17). Thus, investigating hippocampal connectivity using this anterior and posterior hippocampal distinction has the potential to further elucidate network changes in TLE and how these hippocampal parcels might relate to memory impairments.

This anterior-posterior parcellation has been applied in a prior study of functional connectivity in individuals with TLE. Voets et al. (5) examined the strength of the timeseries correlation between each voxel in the hippocampus to target masks constructed from regions known to be connected to the anterior and posterior hippocampus in the healthy brain. If a voxel showed stronger correlation to the “anterior memory mask” (composed of entorhinal cortex, orbitofrontal cortex, and temporal pole) compared to the “posterior memory mask” (composed of parahippocampal gyrus, lingual/fusiform gyrus, dorsolateral pre-frontal cortex, posterior cingulate cortex, precuneus, and thalamus) then it was assigned to the anterior hippocampus. Conversely, a voxel that showed stronger correlation to the posterior mask was labeled as the posterior hippocampus. Using this technique, they demonstrated that patients and healthy controls showed a similar anterior and posterior division that was split along the long axis of the hippocampus. Further, combining individuals with left lateralized TLE (LTLE) and those with right lateralized TLE (RTLE), revealed that deviations in resting connectivity strength were associated with material-specific memory impairments; i.e., verbal memory impairment in left TLE and visuospatial memory impairment in right TLE. Impaired, relative to intact, memory was associated with both increased connectivity strength between the ipsilateral anterior hippocampus and entorhinal cortex and decreased connectivity strength between the contralateral posterior hippocampus and posterior cingulate cortex.

While these are very important findings linking disrupted connectivity and memory deficits in TLE, there are several assumptions underlying these analyses that may be challenged. First, they used anatomical masks to define their anterior and posterior memory network. Patterns of inter-regional correlation, however, are not strictly circumscribed to the gyral anatomy of most atlases and, in fact, broad networks often cross over and between anatomical boundaries (18). Second, their method for labeling voxels in the hippocampus as either anterior or posterior was somewhat crude. They labeled a voxel as an anterior voxel if it demonstrated greater correlation to the mean time series of the whole anterior memory network mask compared to the posterior memory network mask. This assumes a certain level of homogeneity of correlation of these anterior and posterior memory networks, ignoring connectivity patterns in favor of magnitudes averaged across large networks, which may not be valid especially for networks defined with anatomical boundaries. We submit that identifying abnormalities in connectivity via a data-driven approach with fewer assumptions provides a reliable, complementary solution. An elegant approach drawn from the literature involves parcellation of hippocampus based on a *k*-means clustering of the voxel connectivity patterns to the whole brain as has been done in the thalamus (19), cingulate cortex (20), and hippocampus (11).

Thus, the aim of this study was to use resting state functional connectivity and *k*-means clustering to parcellate the hippocampus of healthy controls and patients with TLE. We further sought to investigate whether the connectivity strength of resulting parcels was related to memory ability. The posterior cingulate cortex (PCC) and the anterior medial pre-frontal cortex (mPFC) are the two core hubs of the DMN (14, 21). Based on our own findings of differential connectivity of anterior and posterior hippocampus to these hubs in the healthy brain (11), together with the patterns indicated in the study by Voets et al. (5), we examined the correlation in resting state BOLD activity between anterior hippocampus and mPFC, and between the posterior hippocampus and PCC. We interrogated whether these correlation patterns would relate to memory ability, highlighting the role of differential anterior and posterior hippocampal connectivity as potential indicators of memory network integrity. Consistent with previous literature on hippocampal functional specialization (9), we hypothesized that the *k*-means clustering would produce anterior and posterior hippocampal clusters. Given that atrophy and gliosis in MTS is biased toward the anterior hippocampus, we also expected that there might be greater alterations in connectivity in anterior hippocampal clusters in people with MTS. Finally, we predicted that individuals exhibiting weaker correlation between the anterior and posterior hippocampal parcels and the respective primary hubs of the DMN (i.e., mPFC and PCC), would have worse material-specific memory deficits.

## Methods

### Participants

Forty-six adult patients with pharmacologically intractable unilateral TLE were recruited from the Epilepsy Clinic at Toronto Western Hospital. Twenty-three patients presented with RTLE and 23 presented with LTLE. Continuous recording of scalp EEG and video monitoring during an inpatient evaluation in our epilepsy monitoring unit were used to determine seizure focus. Nineteen neurologically healthy control subjects were recruited to serve as comparison for our patient sample for to identify alterations in resting-state fMRI networks. All controls gave prospective written informed consent. Prospective written informed consent was obtained from a subset of the patient group, while permission for retrospective analysis of clinical data (both neuropsychological and resting-state fMRI) was obtained from the University Health Network Ethics Board for a group of participants who were scanned prior to the current ethics protocol implementation.

### Neuropsychological Testing

A comprehensive neuropsychological battery was administered to patients that included assessment of intelligence, learning/memory, processing speed, and verbal and visuospatial functioning. The battery included the following measures: Wechsler Abbreviated Scale of Intelligence, Warrington recognition memory test for faces, Rey visual design learning test, conditional associative learning test, Warrington recognition memory test for words, and Rey auditory verbal learning test. For each patient, we transformed eight raw scores from these tests into summary factor scores using previously estimated factor loadings from a principle component analysis (PCA) performed by St-Laurent et al. (22). In brief, St-Laurent et al. (22) performed a PCA on neuropsychological scores from a group of individuals with TLE, similar to the current cohort. The PCA revealed three significant components which the authors characterized as reflecting IQ, visuospatial memory, and verbal memory based on the loading of the individual neuropsychological tests to each factor. These factor scores were able to (1) discriminate between patients with right and left TLE and (2) reliably predicted the degree of material-specific memory change following anterior temporal lobe resection (22). Thus, by transforming the raw scores from the neuropsychological assessment of patients in the current study into these summary factor scores we are able to assess a more reliable representation of core abilities than single test scores. The IQ factor reflected loadings from verbal IQ and performance IQ from the Wechsler Abbreviated Scale of Intelligence (23).

The visuospatial memory (VSM) factor was primarily based on loadings from correct responses on the Warrington recognition memory test for faces (RMF), total recall across trials one through five on the Rey visual design learning test (RVDL), and number of trials to criterion for the conditional associative learning test (CAL). The RMF test involves a study period in which 50 faces are viewed, followed by a recognition test in which subjects are asked to make a forced choice recognition decision between previously studied faces and lures (24). The RVDL consists of a study session for 15 abstract visual line designs followed by an immediate recall session in which subjects are asked to draw the previously encountered visual designs (25). This is repeated five times. Finally, the CAL consists of having patients learn a one-to-one association between four cards and four spatial locations through trial-and-error (26).

The verbal memory (VM) factor was based on loadings from correct responses on the Warrington recognition memory test for words (RMW), total recall (RAVLT-tot) over five study-test trials from the Rey auditory verbal learning test (RAVLT) and percent retention (RAVLT-ret) from the RAVLT. The RMW test consists of a study session for 50 words followed by a delayed forced choice recognition test between lures and studied words (24). The RAVLT consists of a study session for 15 words followed by an immediate free recall period. This is repeated five times. Percent retention for the RAVLT is calculated by observing the percentage of words retained from the fifth session on a 20-min delayed recall trial (27).

### Statistical Analysis of Behavior and Demographics

To compare clinical, demographic, and behavioral measures, we used SPSS 21 (Chicago, IL). One-way ANOVA's were used to investigate group differences in age and education. Chi-squared tests were used to investigate group differences in sex distribution, and, between the TLE groups, presence of MTS and presence of other lesions. Fisher's exact tests were used to examine group differences in handedness and, between the TLE groups, laterality of language dominance. Student's *t*-test were used to investigate differences in age of onset, duration of epilepsy, verbal memory, visual memory or IQ between the TLE groups.

### MRI Acquisition

A high-resolution 3D anatomical scan was collected on a 3T Signa MR system (GE Medical Systems, Milwaukee, WI, USA) for normalization to standard MNI space for each subject (T1-weighted sequence, FOV 220 mm, 146 slices, flip angle = 12°, 256 × 256 matrix, resulting in voxel size of 0.86 × 0.86 × 1.0). Resting state fMRI (T2^*^-weighted) scans were acquired with an echo-planar pulse imaging (EPI) sequence (FOV 240 mm, 28–32 slices depending on head size, TR = 2,000 ms, TE = 25 ms, 64 × 64 matrix, 3.75 × 3.75 × 5 mm voxels, for 180 volumes). During resting state scans, subjects were instructed to lie still, and “not to think about anything in particular,” with their eyes closed.

### Functional MRI Pre-processing

Preprocessing was performed using SPM8 (http://www.fil.ion.ucl.ac.uk/spm/software/spm8), a toolbox running in MATLAB 7.9 (Mathworks). Anatomical and functional images were reoriented so that the origin falls on the anterior commissure. The functional images were then co-registered to the anatomical image before undergoing realignment and unwarping for motion correction. Anatomical images for each subject were segmented into gray matter, white matter and cerebral spinal fluid (CSF) and normalized into standard MNI space. Functional images were then normalized to standard space using the parameters from the anatomical transformation. Smoothing varied for the *k*-means clustering analysis and the group comparisons analysis. For the *k*-means clustering analysis, two separate threads of processing then occurred with one thread undergoing spatial smoothing with a 4-mm full-width half-max (FWHM) Gaussian kernel and the other having no spatial smoothing performed. The reason for these separate smoothing parameter threads will be described below. For group comparisons, we smoothed the data with an 8-mm FWHM Gaussian kernel. Next, using the Artifact detection toolbox (28), fluctuations in global signal >3 standard deviations, translational motion >1 mm, and rotational motion >0.05 radians were identified and regressors were created to exclude these potentially confounding sources of variance. Finally, in the Conn toolbox (28), temporal filtering was performed to exclude low (<0.008 Hz) and high (>0.09 Hz) frequency fluctuations, and a CompCor (29) was used to exclude measures of physiological noise by regressing out the top five components of a principle components analysis from the white matter and CSF masks produced from the SPM8 segmentation. The filtered and corrected images were used for subsequent analyses.

### *k*-Means Clustering

To identify functionally distinct sub-regions of the hippocampus, we performed a functional connectivity-based parcellation using a *k*-means clustering algorithm. First, left and right whole-hippocampus masks were defined using the Harvard-Oxford subcortical structural probabilistic atlas in FSL. For each of the left and right hippocampus region of interest (ROI), we probed the functional organization of the ROI by testing the correlation between the time series of each voxel within an ROI and the time series of every other gray matter voxel in the brain. We therefore used the individual subject segmented normalized gray matter images to isolate the voxels for which the time series correlation to the hippocampal ROIs would be computed. Critically, while the whole-brain gray matter mask was minimally smoothed with a 4-mm FWHM Gaussian kernel (see above), the hippocampal voxel time series were not smoothed, to ensure that spatial adjacency in the clustering results was minimally attributable to spatial correlation between neighboring hippocampal voxels.

For each subject, we computed the Pearson's correlation coefficient between the time series of a given voxel within a hippocampal ROI and every other voxel in our whole-brain gray matter mask. This resulted in a whole-brain gray matter statistical map of correlation coefficients for each hippocampal voxel (i.e., that voxel's “functional connectivity profile”). A second-order correlation matrix of each hippocampal voxel's similarity in functional connectivity profiles was computed for each subject. We then averaged the second-order, within-ROI correlation matrices across participants after sorting the voxel sequences along the matrix dimensions identically and performed *k*-means clustering on the group level second-order correlation matrix with a *k* = 2 parcellation. The squared Euclidean metric was used to define distance between clusters, and cluster centroid values were estimated using the *k*-means ++ algorithm implemented in MATLAB. This procedure places random initial seeds for the analysis, and converges quickly to minimize within-cluster, point-to-centroid distance iteratively. We specified a max of 100 iterations for convergence, and 25 replications with random initial seeds were conducted to reduce the probability of convergence onto local minima. The correlation matrix of functional connectivity profiles within each ROI was sorted according to the cluster labels derived from the *k*-means cluster analysis. These steps are illustrated in Figure S1. The results also produced a cluster label for each voxel in the hippocampal ROI which were then projected back to standard brain space at the group level to create anterior and posterior hippocampal masks. The resulting clusters were visually examined (by author AB), and voxels which were located on the periphery of the hippocampus and isolated from other voxels in the cluster assignment were identified and removed, as these voxels were likely misclassified. This resulted in 2/522 voxels being excluded in the Left Hippocampus of Controls, 8/522 voxels being excluded in Left Hippocampus of the RTLE group, and 27/533 voxels being excluded in the Right Hippocampus of the RTLE group.

### Region of Interest Analysis

To interrogate the functional connectivity differences between the TLE and healthy control groups, we used the resulting masks from the *k*-means clustering analysis as regions of interest for the subsequent analyses. The mean time course from each ROI was correlated with the smoothed data from every other voxel in the brain. These correlations were then transformed using a Fisher's z transformation. The resulting individual subject maps were entered into a group level between-subject analysis, to examine differences in voxel-wise whole brain connectivity of the anterior and posterior hippocampus from the left and right hemisphere. Analyses were performed separately for the LTLE group and RTLE group. We contrasted the whole brain connectivity maps from each *k-*means cluster between the control group and the TLE groups. Resulting contrast maps were corrected using permutation analysis with 5,000 permutations at *p* < 0.005 cluster defining threshold, and false discovery rate corrected at *p* < 0.05. Given that previous research had shown an increase in left anterior hippocampal connectivity to the entorhinal cortex (5), and posterior hippocampal connectivity to the parahippocampal gyrus, we also explored these connections using a small volume correction with the entorhinal cortex mask from the Juelich histological atlas and the Harvard-Oxford parahippocampal gyrus mask, thresholded at 35% [the same mask used by Voets et al. (5)]. Years of education was entered for each subject and investigated as a covariate of no interest as this differed by group (see below). Following this, we sought to see if the presence of MTS was driving the connectivity differences between TLE and control groups. To that end, we extracted the peak connectivity from the resulting significant voxel clusters that differed between the TLE groups compared to the controls and performed a randomization test between the MTS subgroup and no-MTS subgroups with 5,000 permutations using the mult_comp_perm_t2 function in MATLAB (Groppe, 2015, Toronto).

To examine the relationship between the altered hippocampal connectivity and memory performance, we used the PCC and mPFC seeds reported by Andrews-Hanna et al. (14). The left PCC seed is located at x = −8, y = −56, z = 26, while the right PCC seed is located at x = 8, y = −56, z = 26, with both having an 8-mm sphere drawn around the center points. The left mPFC seed is located at x = −6, y = 52, z = −2, and the right is located at x = 6, y = 52, z = −2 with 8-mm spheres drawn around the center points. The mean time course of each hippocampal cluster was extracted and correlated with the mean time course of the corresponding PCC or mPFC seed. These correlation coefficients were then Fisher z-transformed and the resulting z-scores were correlated with memory scores using the verbal and visual memory factor scores using SPSS 21 (Chicago, IL). We were specifically interested in how connectivity of the epileptogenic hippocampus related to material-specific memory (verbal memory in LTLE and visual memory in RTLE). Left language dominance is thought to be less consistent in TLE and also appears to play a role in verbal memory (30–32). Therefore, we examined the brain-behavior correlations in individuals with left language dominance. Results for the full analysis are available in Supplementary Table 1.

## Results

### Demographic Data

There were no differences between the three groups in terms of age, *F*_(2, 62)_ = 0.51, *p* = 0.6 or handedness, Fisher's exact test, *p* > 0.5. There was a significant difference between the three groups in terms of education, *F*_(2, 62)_ = 9.7, *p* < 0.01, with healthy controls having greater education than both the LTLE and RTLE group using a Bonferroni *post-hoc* test. There was a different proportion of male and females between the three groups, χ^2^(2, *N* = 65) = 10.2, *p* = 0.006. Specifically, there was a difference between the LTLE and RTLE group in terms of sex distribution, χ^2^(1, *N* = 46) = 8.7, *p* = 0.003. There were no differences in age of onset, duration of epilepsy, verbal memory, visual memory or IQ, between the LTLE and RTLE groups, all *t* <1.5, *p* > 0.15, nor were there any differences between patient groups in presence or absence of MTS, distribution χ^2^(1, *N* = 46) = 0.37, *p* = 0.5, in the presence of other lesions, χ^2^(1, *N* = 46) = 1.1, *p* = 0.3, or in language dominance using Fisher's exact probability test, *p* = 0.1. Demographic information and neuropsychological performance are reported in Table 1.

**Table 1:** Patient demographic data.

	**Controls**	**RTLE**	**LTLE**
*N*	19	23	23
Age, y (SD)	34 (22–59)	36.9 (18–58)	37.6 (24–53)
Education, y (SD)	18 (13–26)	14.2 (8–22)	14.2 (11–18)
Sex, M/F	11/8	17/6	7/16
Handedness, R/L/BI	17/2/0	22/1/0	20/2/1
Language dominance, R/L/BI	–	0/23/0	1/20/2
Disease duration, y (SD)	–	15.6 (1–48)	18.4 (3–46)
Onset of seizures, y (SD)	–	21.0 (0–51)	19.2 (0.67–50)
Presence of MTS, Yes/No	–	15/8	13/10
Other lesions	–	3	1
Verbal memory factor	–	0.23(1.2)	0.19 (1.1)
Visual memory factor	–	−0.20 (1.1)	0.20 (0.77)
IQ Factor	–	−0.14 (1.2)	0.36 (1.0)

*RTLE, right temporal lobe epilepsy; LTLE, left temporal lobe epilepsy; y, years; SD, standard deviation; M, male; F, female; R, right; L, left; BI, bilateral; IQ, intelligence quotient. Characterization of MTS and other lesions was based on radiology (3T MRI protocol). In the RTLE group, one individual had a right amygdala ganglioglioma, one individual had a right amygdala dysembryoplastic neuroepithelial tumor, and one had a right amygdala harmartoma. In the LTLE group, one individual had a left amygdala dysembryoplastic neuroepithelial tumor*.

### *k*-Means Clustering

**Figure 1:**
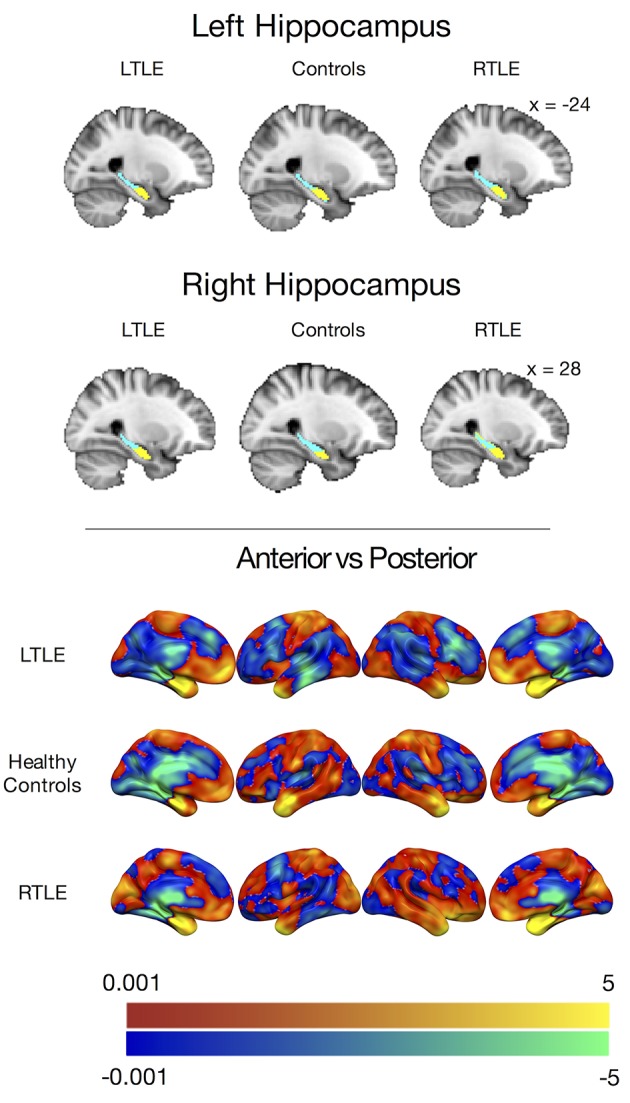
**(Top)** Group level clusters from k-means clustering procedure, projected onto the standard MNI brain showing an anterior (yellow) and posterior (blue) cluster for both the left and right hippocampus derived from patients with left temporal lobe epilepsy, healthy controls, and patients with right temporal lobe epilepsy. **(Bottom)** Anterior hippocampal connectivity contrasted against posterior hippocampal connectivity in people with left temporal lobe epilepsy (LTLE), healthy controls, and people with right temporal lobe epilepsy (RTLE) presented without statistical thresholding. Warm colors indicate anterior > posterior hippocampal connectivity, while cool colors indicate posterior > anterior hippocampal connectivity. The color bars depict *t*-values.

The *k*-means clustering procedure produced visually similar clusters for all three groups in both hemispheres, with anterior and posterior clusters divided along the long axis of the hippocampus. These clusters are displayed in Figure 1. A few voxels along the borders of the hippocampus seemed to misclassify. This was likely due to noisy voxels that may represent white matter or cerebral spinal fluid that were encapsulated by the Harvard-Oxford hippocampal mask. Prior to group level connectivity analysis, these voxels were deleted from the clusters.

To examine the preferential functional connectivity of each of these clusters in the groups, we contrasted the voxel-wise correlations of the left and right anterior hippocampi with the left and right posterior hippocampi (Figure 1). Consistent with previous work and our predictions, all groups showed stronger positive correlations between the anterior hippocampus and the temporal pole, amygdala, and ventral pre-frontal cortices, including the mPFC while the posterior hippocampal parcels showed stronger positive correlations with the parahippocampal gyrus and thalamus. The LTLE and healthy control groups also showed stronger positive correlations between the posterior hippocampal parcels and the posterior medial regions, such as the PCC, but this was not the case for the RTLE group.

### Group Connectivity Differences

When seeding from the left anterior hippocampal cluster, the LTLE group showed reduced connectivity to the parahippocampal cortex bilaterally, reduced connectivity to midline parietal and pre-frontal cortex, bilaterally, and reduced connectivity to the left angular gyrus compared to the healthy control group. There were no areas of increased connectivity with the left anterior hippocampus in LTLE compared to controls when examining the whole brain. Targeted analysis found increased connectivity between the left anterior hippocampus and the left entorhinal cortex, centered around xyz = −24, −14, −32, *t*_(41)_ = 3.9, *p* < 0.001.

**Figure 2:**
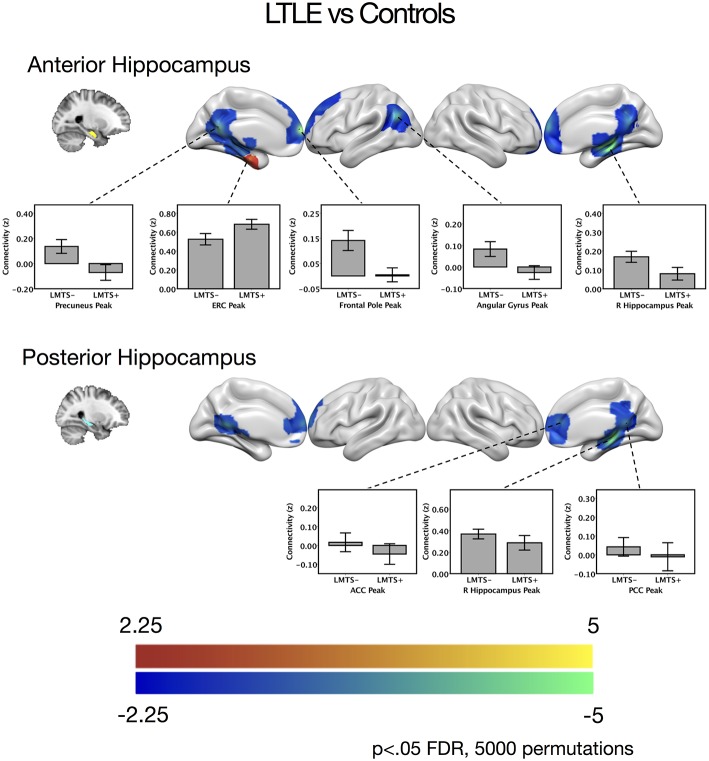
Contrast maps of differences in connectivity between patients with left temporal lobe epilepsy compared to controls, seeding from either the anterior hippocampal seeds (top) or the posterior hippocampal seeds (bottom), thresholded at *p* < 0.05 FDR cluster correction, using 5,000 permutations. Areas of increased connectivity in LTLE, depicted in red, were only significant using a small volume correction in the entorhinal cortex. Areas of reduced connectivity in LTLE compared to controls are shown in cool colors. Bar plots depict mean group connectivity and standard error at voxels of peak difference between the LTLE group and controls, with the LTLE group separated into those with mesial temporal sclerosis (LMTS+), and those without (LMTS−). These peaks are presented below in Table 2. The color bars depict *t*-values. ACC, anterior cingulate cortex; ERC, entorhinal cortex; PCC, posterior cingulate cortex; R, right.

A similar pattern of reduction was seen for the left posterior hippocampal cluster, with reduced connectivity to midline parietal and pre-frontal cortex, bilaterally, and reduced connectivity to the right medial temporal cortex. There were no areas of increased connectivity for the posterior hippocampus even when using a small volume correction with the parahippocampal mask from the Harvard-Oxford atlas, as was used by Voets et al. (5). There were no connectivity differences between the LTLE group and healthy controls for either the anterior or posterior right hippocampal seeds. These results are displayed in Figure 2 and peak coordinates for these analyses are presented in Table 2. We sought to determine whether the presence of MTS influenced connectivity differences, and thus extracted the peak connectivity values from each significant cluster. When we compared the connectivity between those with MTS compared to those without MTS we found that the patients with MTS had lower connectivity to DMN regions (Right hippocampus: *p* = 0.03; Frontal pole: *p* = 0.003; Left angular gyrus: *p* = 0.02; Precuneus: *p* = 0.009), and higher connectivity to the left entorhinal cortex (*p* = 0.03), compared to the patients without MTS. In the left posterior seed, however, there were no significant connectivity differences between patients with and without MTS (*p* > 0.17).

**Table 2:** Cluster regions, peak coordinates, test statistic, and cluster size for connectivity differences between people with LTLE and healthy controls.

**Region**	**Hemisphere**	**x**	**y**	**Z**	**T**	**Cluster size (voxels)**
**LEFT ANTERIOR HIPPOCAMPUS SEED**						
Ant Hippocampus	R	24	−16	−16	−7.07	1,418
Angular gyrus	L	−50	−66	28	−4.38	1,462
Precuneus	B	−12	−52	16	−5.17	2,613
Frontal Pole	B	−10	62	14	−5.65	3,140
Entorhinal cortex	L	−24	−14	−32	3.9	207
**LEFT POSTERIOR HIPPOCAMPUS SEED**						
Post hippocampus	R	26	−28	−14	−5.62	2,680
Posterior cingulate	B	12	−46	12	−4.24	
Anterior cingulate	B	4	44	12	−4.25	1,580

*Ant, anterior; B, bilateral; L, left; Post, posterior; R, right. Coordinates are presented in MNI space*.

**Figure 3:**
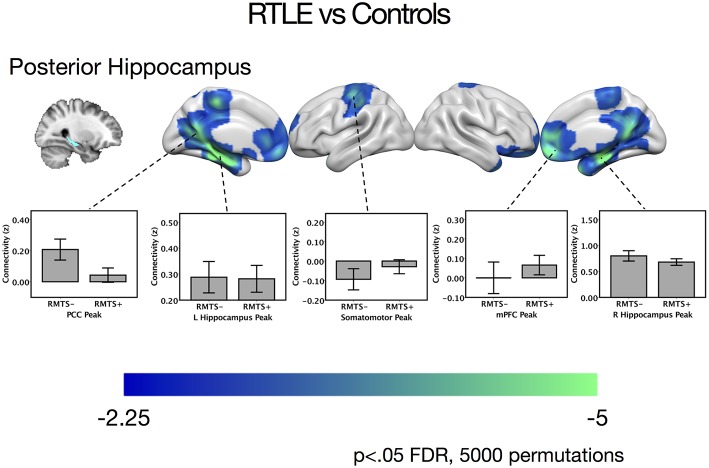
Contrast maps of differences in connectivity between patients with right temporal lobe epilepsy compared to controls, seeding from the right posterior hippocampal seed, thresholded at *p* < 0.05 FDR cluster correction, using 5,000 permutations. Areas of reduced connectivity in RTLE compared to controls are shown in cool colors. Bar plots depict connectivity at areas of peak difference between the RTLE group and controls, with the RTLE group separated into those with mesial temporal sclerosis (RMTS+) and those without (RMTS−). These peaks are presented below in Table 3. The color bars depict t values. L, left; mPFC, medial pre-frontal cortex; PCC, posterior cingulate cortex; R, right.

There were no significant differences between the RTLE group and the healthy control group when seeding from the right anterior hippocampal region. As this was surprising, we have provided maps for this contrast using a relaxed cluster defining threshold of *p* < 0.05, corrected using FDR at *p* < 0.05 and 5,000 permutations in Figure S2, and examined effect sizes in Supplementary Methods and Results. When seeding from the right posterior hippocampus, there was reduced connectivity to bilateral medial temporal cortex, right temporal pole, bilateral midline parietal and pre-frontal cortex, right lateral orbitofrontal cortex, and right somatomotor cortex in the RLTE group compared to controls. There were no areas of increased connectivity for the right posterior hippocampus, even when using a small volume correction in the parahippocampal gyrus. There were no connectivity differences between the groups for either the contralateral (left) anterior or posterior hippocampal seeds. These results are displayed in Figure 3 and peak coordinates for these analyses are presented in Table 3. Again, when examining the voxels of peak differences, there were no differences in connectivity for the posterior epileptogenic hippocampus for the patients with MTS compared to those without (*p* > 0.05).

**Table 3:** Cluster regions, peak coordinates, test statistic, and cluster size for connectivity differences between people with RTLE and healthy controls.

**Region**	**Hemisphere**	**x**	**y**	**z**	**T**	**Cluster size (voxels)**
**RIGHT POSTERIOR HIPPOCAMPUS SEED**						
Ant hippocampus	R	26	−18	−16	−10.03	18,726
Ant hippocampus	L	−24	−18	−22	−9.44	
Temporal Pole	R	34	12	−40	−6.04	
mPFC	B	2	52	−10	−5.32	
Posterior cingulate	B	−10	−50	4	−5.32	
Precentral	B	−6	−30	54	−5.28	
Precentral	L	−36	−18	56	−5	

*Ant, anterior; B, bilateral; L, left; mPFC, medial prefrontal cortex; R, right. Coordinates are presented in MNI space*.

### Hippocampal Connectivity and Memory

**Figure 4:**
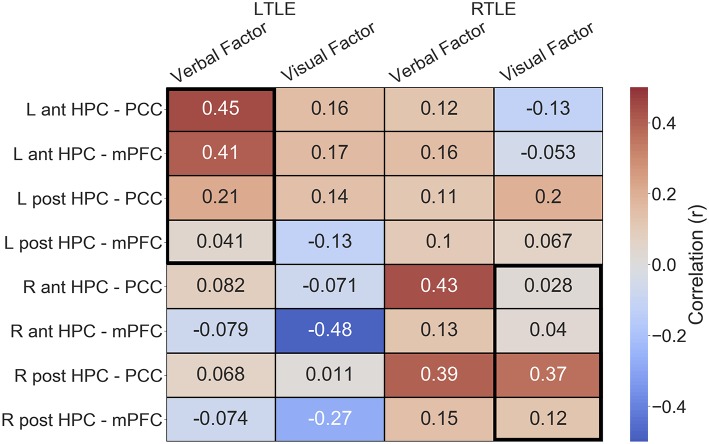
Correlation matrix depicting the relationship of functional connectivity to verbal and visual memory in the LTLE and RTLE groups. Thick black outlines delineate relationships of interest. Ant, anterior; L, left; LTLE, left temporal lobe epilepsy; HPC, hippocampus; mPFC, medial pre-frontal cortex; PCC, posterior cingulate cortex; Post, posterior; R, right; RTLE, right temporal lobe epilepsy.

Given the alterations in connectivity of the epileptogenic hippocampal clusters, we sought to examine how connectivity of these areas to DMN hubs related to verbal and visual memory in people with LTLE and people with RTLE, respectively. In the LTLE group, we observed a medium-sized positive relationship between the verbal memory factor score and functional connectivity of the left anterior hippocampus to PCC, *r*_(18)_ = 0.45, *p* = 0.02, similar to previous reports that examined the whole hippocampus [(6): *r* = 0.72]. A medium-sized positive relationship was also seen between the verbal memory factor score and functional connectivity of the left anterior hippocampus to mPFC, *r*_(18)_ = 0.41, *p* = 0.04. These relationships, however, do not survive corrected statistical thresholds (*p* < 0.0125). The left posterior hippocampus had numerically weaker, and non-significant relationships, as shown in Figure 4. While we made no predictions about brain-behavior correlations with visual memory in LTLE, or with regards to the contralateral hippocampal connectivity, we present the full correlation matrix for display purposes.

In the RTLE group, we observed a medium-sized positive relationship between the visual memory factor score and functional connectivity of the right posterior hippocampus to PCC, *r*_(21)_ = 0.37, *p* = 0.04, similar to previous reports that examined the whole hippocampus [(6): *r* = 0.73]. This relationship also does not survive corrected statistical thresholds (*p* < 0.0125). The other connections of interest showed minimal relationships to visual memory, all *r* < 0.13.

We also observed several other relationships, that we were not specifically anticipating, that had comparable strength. We observed a negative relationship between the visual memory factor and functional connectivity of the right anterior hippocampus to mPFC, *r*_(18)_ = −0.48, in the LTLE group. We also observed that connectivity of the PCC to the epileptogenic hippocampus, both anterior and posterior clusters, was related to verbal memory in the RTLE group (anterior: *r*_(21)_ = 0.43; posterior, *r*_(21)_ = 0.39).

## Discussion

Using a *k*-means clustering procedure, we were able to segment the left and right hippocampus into anterior and posterior divisions in individuals with left and right TLE and healthy controls. This demonstrates that the functional connectivity fingerprints of the hippocampal voxels are sufficiently distinguished along the long axis in the patient population, regardless of the effects of temporal lobe epilepsy. At the group level when these segments are compared directly, the anterior clusters showed greater connectivity to the temporal pole, amygdala and ventral pre-frontal cortices, while the posterior clusters showed increased connectivity to the parahippocampal gyrus and thalamus across all groups. Between group contrasts of functional connectivity showed significant reductions in connectivity between the epileptogenic hippocampus and DMN regions in both LTLE and RTLE groups compared to healthy controls, but in the RTLE group this was limited to the posterior hippocampus at the reported thresholds. In areas of the DMN regions showing reduced connectivity, the LTLE patients with MTS demonstrated notably aberrant connectivity relative to the patients without MTS, restricted to the anterior epileptogenic seed, which suggests that the presence of structural pathology exacerbates network alterations, partially supporting our hypothesis that MTS pathology would preferentially affect anterior hippocampal network changes.

Due to the growing consensus regarding the specialization of function in the long axis of the hippocampus (9), previous work has attempted to characterize functional connectivity differences between the anterior and posterior hippocampus (11, 12, 33, 34). Previous characterizations probed the functional connectivity using varying methods such as delineating the hippocampus by anatomical landmarks (33), placing seed spheres along the long axis of the hippocampus (12), examining connectivity slice by slice along the y-axis of the hippocampus (34), and by examining the connectivity of seed masks resulting from hippocampal parcellation using structural connectivity via DTI (11). This previous work has consistently shown that the anterior hippocampus tends to show greater functional connectivity to perirhinal, ventral-temporal, lateral temporal, and temporopolar cortex, while the posterior hippocampus tends to have greater functional connectivity to the parahippocampal gyrus, retrosplenial, and lateral parietal cortex. The resulting parcels from our parcellation results also showed these functional connectivity biases which provided reassurance that our subsequent analyses using these parcels was targeting meaningful networks supported by the literature.

Our parcellation findings are in agreement with previous work by Voets et al. (5) who similarly distinguished anterior and posterior clusters in the hippocampus using functional connectivity. Their methods involved giving a binary label to a voxel based on whether its magnitude of connectivity was arithmetically larger to either an anterior or a posterior memory network mask. This method required specification of regions *a priori*, ignored patterns of connectivity and instead focussed merely on average connectivity magnitude. We refined the analytic approach here, nonetheless providing convergent evidence that the functional connectivity patterns of the hippocampal voxels form an anterior and posterior compartment regardless of the effects of longstanding epileptic seizures arising from the medial temporal lobe.

In our functional connectivity analysis, we found reduced connectivity between the epileptogenic hippocampus and DMN regions in TLE compared to healthy controls, similar to previous studies. More specifically, we observed that the epileptogenic posterior hippocampus had reduced connectivity to the PCC and mPFC, DMN hubs, in both TLE groups compared to healthy controls. Additionally, connectivity between hippocampus and other DMN nodes was also reduced, with significant findings for right posterior hippocampus to temporal pole in the right TLE group. In LTLE, the reductions overlapped considerably between the anterior and posterior left hippocampus, despite observations from previous research that these parcels have different preferred connectivity patterns (5, 11, 12). However, it is important to note that, while these anterior and posterior parcels will have connectivity preferences, both tend to connect with similar DMN regions in the healthy brain (11). The only other study comparable to ours, by Voets et al. (5) demonstrated a somewhat different pattern of connectivity alterations associated with TLE. While they also report a decrease in connectivity between posterior hippocampus and PCC, they further report significant increases in connectivity for both TLE groups (posterior hippocampus to parahippocampal gyrus) and for the left TLE group (anterior hippocampus to entorhinal cortex). While we did replicate their latter finding, using small volume correction, we did not observe the former increase or indeed any other increases in connectivity in TLE groups compared to controls. Some of these differences may be due to methodological factors such as whole-brain analysis vs. a priori regions of interest. There is sparse literature on resting-state connectivity from the hippocampus in TLE, but one other paper (3) found similar decreases to our work together with increases in primarily subcortical areas none of which are associated with DMN. Clearly more work needs to be done to ascertain the nature of pathological changes in this network.

Altered network connectivity may be the result of interictal epileptic discharges observed in TLE which are known to disrupt the functioning of the DMN (35), leading to the deterioration seen in the cingulum bundle that connects the medial temporal lobe to the PCC and medial pre-frontal cortex (36). In patients who had left MTS, connectivity reductions of the left anterior hippocampus to the DMN were larger than in patients who did not have MTS, which supports research showing greater structural connectivity declines in MTS for regions that connect the DMN such as the cingulum and fornix (37–39). In the RTLE group, we found reductions in connectivity of the right posterior hippocampus to DMN regions, but contrary to expectation, there were no significant connectivity differences in the right anterior hippocampus between the RTLE group and healthy controls. When using a more liberal cluster defining threshold of *p* < 0.05 with FDR correction using 5,000 permutations, we did see reduced connectivity of the right anterior hippocampus restricted to diffuse DMN regions, and, thus, our null finding may relate to our power to detect this effect. When calculating statistical power to detect the estimated effect size for anterior hippocampus, we found that our sample was underpowered to detect an effect in the RTLE group (Supplementary Results), which appears to reflect a difference in the impact of MTS on anterior connectivity. This disparity in effect sizes between the LTLE and RTLE groups fits with reports of greater pathology in left sided TLE compared to right, in terms of white matter structure (40), and widespread gray matter structure (41). Further, white matter imaging with DTI has also shown that there is a stronger correlation between the integrity across white matter tracts in LTLE compared to healthy controls and RTLE (42). Higher integrity correlations between tracts such as the fornix and cingulum bundle may suggest a shared underlying process that alters the white matter integrity, such as seizure activity which may propagate farther in LTLE. These previous convergent findings speak to the possibility that seizure-related disruptions propagate more readily in LTLE.

When interrogating the relationship between memory and hippocampal connectivity, we found that poorer material-specific memory was modestly associated with weaker connectivity between the epileptogenic hippocampus and major hubs of the DMN, consistent with previous work (6). While our findings did not survive statistical correction for multiple comparison, we below discuss the pertinent literature and speculate on the role of the hippocampus for memory in TLE. The mPFC and PCC are primary hubs of the DMN (14), a network which shows strong overlap with autobiographical memory regions (43), and have been implicated in episodic memory in TLE (4). The mPFC and PCC both have many proposed roles in memory, but both are implicated in contextual representation (44, 45) and episodic retrieval (46). At a global level, weaker connectivity to these hubs may indicate a reduced ability to bind information to the encoding context or an inability to reinstate the original context at retrieval. The anterior hippocampus is thought to communicate with regions such as the anterior temporal pole, and perirhinal cortex (11, 12) which represent concepts and item level features, respectively (47, 48). Thus, reductions in connectivity between the anterior hippocampus and these DMN hubs may indicate a reduced ability to bind conceptual verbal information with contextual information, as was seen in the LTLE group. On the other hand, the posterior hippocampus has stronger resting connections to ventral visual regions such as the fusiform, lingual gyrus and parahippocampus (5, 12), which represent visual and configural information, and also actively communicates with these regions during vivid elaboration of autobiographical memories (49). In RTLE, the right posterior hippocampus had significantly reduced connectivity to posterior medial regions and mPFC and, as such, greater reductions in connectivity between the right posterior hippocampus and the PCC may indicate a reduced ability to bind visual information and contextual information. This conceptualization will require further experiments to interrogate and may eventually inform theoretical frameworks of hippocampal functioning.

One limitation of the current study is that all patients were taking anti-epileptic drugs during the scanning period and it is difficult to exclude the effect that this may have had on functional connectivity of the brain. It is also possible that undetected interictal epileptiform discharges occurring during either scanning or neuropsychological test sessions could have led to alterations in connectivity. However, individuals with epilepsy rely on these medications in their everyday life, and may also experience interictal discharges, and thus, the state of their brain connectivity as depicted here is a reflection of their day to day experience. Another limitation was that each person's brain was transformed into standard space prior to *k*-means clustering which inherently leads to some signal blurring which could affect the parcellation at cluster boundaries. This step was performed to ensure each *k*-means clustering procedure was sampling from the same number of voxels in order to generate group level masks. While some small amount of smoothing may have occurred during transformation to standard space, we did not smooth with a Gaussian kernel inside the hippocampus, and performed minimal smoothing in the rest of the brain. Our results also produced clusters that replicate previous findings in the literature (5), including our previous work which performed clustering in native space (11). Finally, this study also did not examine whether these measures of connectivity were related to post-surgical memory change. Only a small number of this patient group has had surgery and returned for follow-up neuropsychological evaluation, precluding the possibility for statistical analysis. Future studies will assess whether the connectivity in the anterior or posterior hippocampus to the PCC is related to post-operative memory change as this would help inform clinicians and patients of the risk for cognitive morbidity from the surgery.

In conclusion, we demonstrated for the first time that the hippocampus can be parcellated into an anterior and posterior component based on its functional connectivity fingerprint to the brain and this can be done in both healthy adults and in patients with TLE, suggesting that the hippocampus in TLE retains some preferential connectivity along its long axis. We also show that the epileptic hippocampus has reduced connectivity to the mPFC and PCC, the two key hubs of the DMN, and that this connectivity is modestly related to material specific memory ability, with anterior hippocampal connectivity in left TLE relating to verbal memory and posterior hippocampal connectivity in right TLE relating to visual memory. This aligns with our previous findings that hippocampal to DMN connectivity is a useful marker for memory network integrity in individuals with temporal-lobe epilepsy. Furthermore, future studies would be helpful in identifying whether anterior and posterior biases in connectivity can be related to more specific memory operations impaired in individuals with TLE.

## Ethics Statement

Informed consent was obtained from all subjects in this study, which was approved by the UHN Research Ethics Board.

## Author Contributions

AB: study design, data collection, and analysis and manuscript drafting. VM: *k*-means clustering analysis and manuscript editing. MM: study design, supervision of data collection, and manuscript drafting.

### Conflict of Interest Statement

The authors declare that the research was conducted in the absence of any commercial or financial relationships that could be construed as a potential conflict of interest.
