# Supplementary material for: Parcellation of the Hippocampus Using Resting Functional Connectivity in Temporal Lobe Epilepsy
Source: Front Neurol. 2019 Aug 22;10:920. doi: 10.3389/fneur.2019.00920 (PMC6714062; doi:10.3389/fneur.2019.00920)
Supplement: Supplementary file 1 [file Data_Sheet_1.pdf]

Supplementary Material

1 Supplementary Methods & Results

Given that differences between the RTLE group and Control group only emerged at liberal thresholds in the epileptogenic anterior hippocampus, we suspected that our sample may be too low to detect this effect. We thus calculated effect size using Cohen's *d*, for the connectivity difference of the peak voxel in the posterior medial cortex. We did this for both the RTLE and LTLE groups to be presented as descriptive statistics to allow for interpretation of the null results.

The effect size for the difference in right anterior hippocampus – right PCC connectivity in healthy controls compared to the RTLE group was $d = 0.6$, 95% CI = -0.05–1.25, which corresponds to a statistical power of 47%. The effect size for the difference in left anterior hippocampus – left PCC in healthy controls compared to LTLE was $d = 1.1$, 95% CI = 0.41–1.78, which corresponds to a statistical power of 93%.

2. Supplementary Figures & Tables

Supplementary Table 1. Table of correlations (Pearson's r) depicting the relationship of functional connectivity to verbal and visual memory in the LTLE

	LTLE	
	Verbal Factor	Visual Factor
L ant HPC - PCC	.28	-.05
L ant HPC – mPFC	.39	.13
L post HPC – PCC	.11	.06
L post HPC – mPFC	.10	-.13
R ant HPC – PCC	-.09	-.06
R ant HPC – mPFC	-.07	-.51
R post HPC – PCC	-.03	.04
R post HPC – mPFC	.12	-.27

Supplementary Figure 2.

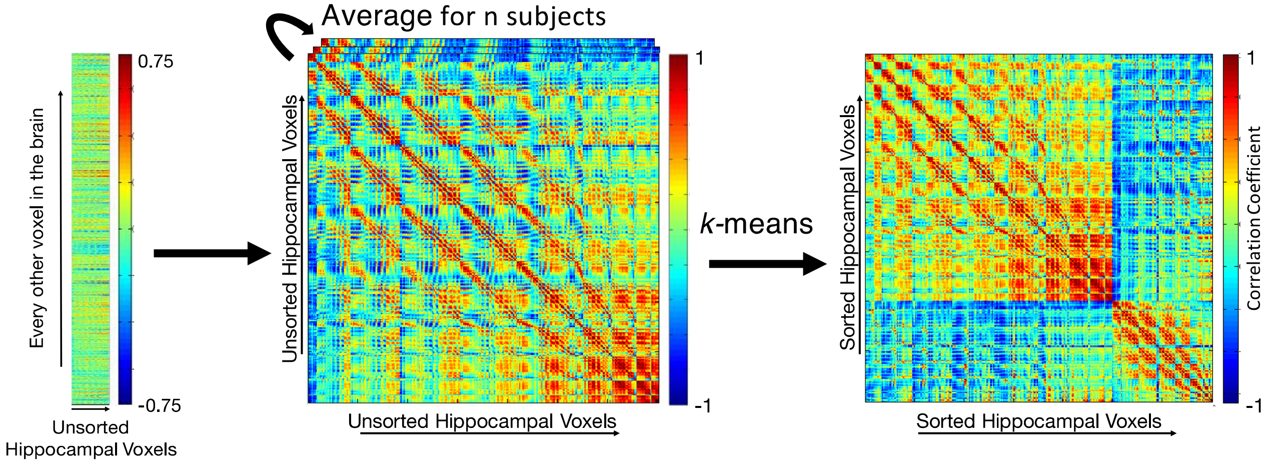

Figure S1. A schematic of the *k*-means clustering procedure. Left: An example of a connectivity matrix for each hippocampal voxel to every other voxel in the brain outside of the hippocampus. Middle: An example of a correlation matrices for each subject in which the whole brain voxel-wise connectivity patterns of each hippocampal voxel are correlated with each other. To make these matrices, each column of the left matrix for each subject is correlated with every other column in a pairwise fashion for an individual subject. Strong correlations between hippocampal voxels indicate that those voxels have similar covariance in their connectivity with the rest of the brain. One matrix is produced per subject and after arranging the voxel sequences in the same order they are averaged together. These subject level matrices were then averaged to make group level hippocampal connectivity matrices. Right: An example of a sorted correlation matrix of whole brain voxel-wise connectivity pattern correlations between each hippocampal voxel following k-means clustering procedure of the group level hippocampal connectivity matrices.

Supplementary Figure 2.

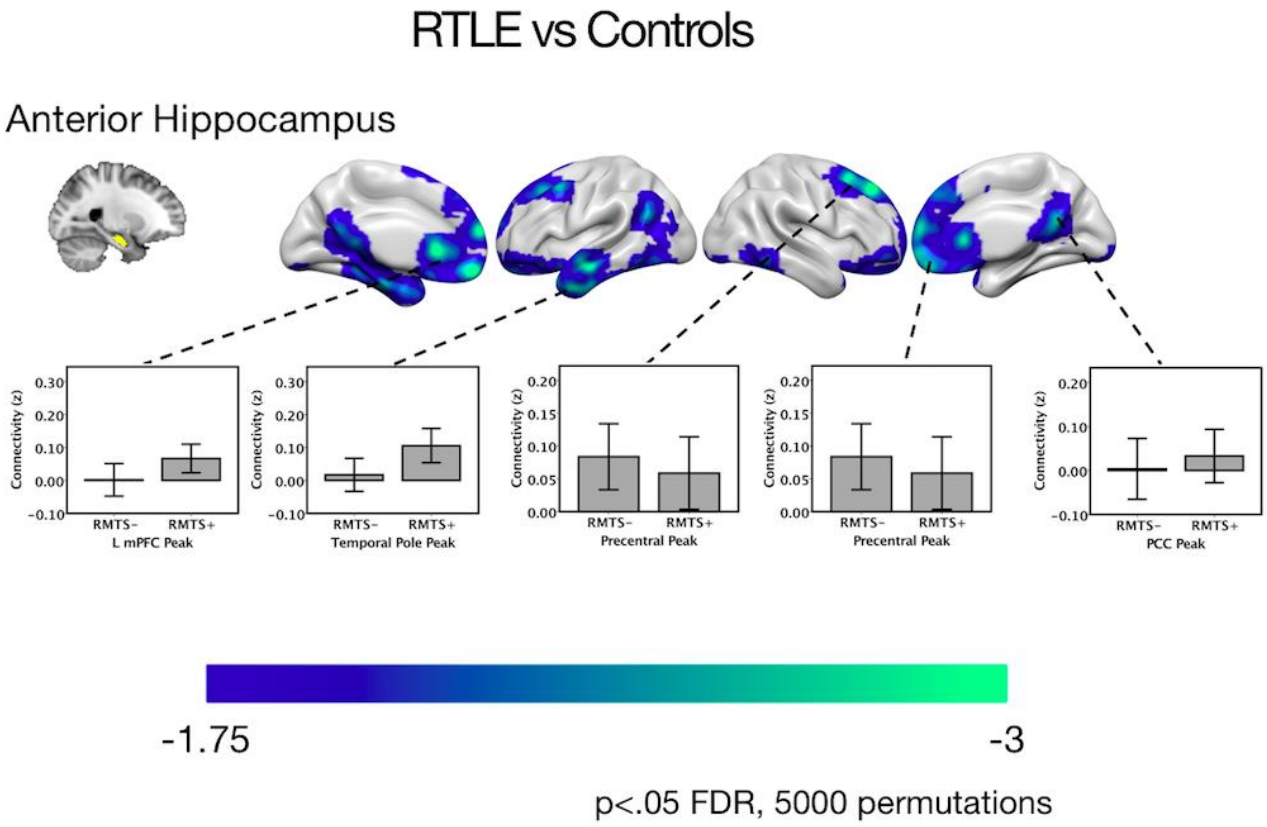

Figure S2. Contrast maps of differences in connectivity between patients with right temporal lobe epilepsy compared to controls, seeding from the right anterior hippocampal seed, thresholded at $p < .05$ FDR cluster correction, using 5000 permutations. Areas of reduced connectivity in RTLE compared to controls are shown in cool colors. Color bar represents *t*-values. Bar plots depict mean connectivity of the RTLE group separated into those with mesial temporal sclerosis (RMTS+), and those without (RMTS-) at voxels of peak difference between the RTLE group and controls. Error bars represent standard error. mPFC, medial prefrontal cortex.
